# Contract Practice

**Published:** 1902-06-21

**Authors:** 


### CONTRACT PRACTICE.

At the present moment when the thoughts of the nation are turned towards the ceremonies and festivities which, according to traditional use are regarded as appropriate on the crowning of a king, it may perhaps seem to savour of indifference to the drift of public feeling if we revert for a moment to more homely matters. Yet in these days of heavy income tax, high rates, and expensive housekeeping, we fancy that many members of our profession are thinking more of their midsummer bills than of the Coronation, and are asking with some anxiety whether they will be able once again to make both ends meet. Indeed, we can imagine that not a few, fretted by the labour of "posting" their books, worried by the long credit exacted by so many of their patients, and irritated by the knowledge that many of these good people never intend to pay at all, are just now looking with more or less envious eyes at the flesh-pots of contract practice; for "Money down" and "No bills" are great temptations to many an exasperated practitioner, even though to enjoy these blessings he might have to submit to the yoke of the "Lodge" and the lash of the "club secretary." Moreover, if one were to regard the matter theoretically, contract practice under proper safeguards might fairly be regarded as even more in accordance with professional ideals than is the bill-making and fee-charging system which prevails among general practitioners. Indeed, when one considers how medical students are brought up, one can hardly wonder that the commercialism of general practice is absolutely revolting to many of them, and one readily understands how it is that even good men sometimes fall victims to the blandishments of those who sing the delights of contract practice, and only find out how bitter is the bait when they have gone too far.

The bitterness of contract work lies first in the fact that when once a man has entered upon it he is no longer his own master, becomes in fact the servant —and the very humble servant, too—of the club or association, while, secondly, it lies in the loss of self-respect which arises from the knowledge that he has entered into an immoral contract. So long as club practice remained a sort of charity, no harm was done. When, however, the club secretary arose, when friendly societies made the medical benefit a "leading article" among their attractions, when they brought within the net of contract practice, at a penny or less a week, people who were quite able to pay, when the club patients instead of being thankful for getting attendance at so cheap a rate began to estimate the value of the services rendered at the ridiculous price they paid for them, then contract practice became an intolerable servitude. This, however, has not been all. A bad system of touting and soliciting for membership has arisen, which is absolutely opposed to all the best traditions of the profession, and thus it has happened that in addition to its other disadvantages participation in contract practice has often involved professional ostracism.

The only remedy for all these evils is that wherever it becomes desirable that any system of contract practice should be established the management of the whole affair should be kept entirely in the hands of the medical profession. This will involve some trouble, no doubt, but it must be done. It is absurd for us to cry aloud to an outside body like the General Medical Council to be kind enough to come down and rule us with mighty threats. The honour of the profession should be in its own hands. If contract practice is to be we should take the trouble to organise it, and we think there is a good deal in the suggestion lately made by Mr. Victor Horsley to the effect that it would be well that such organisation should be effected on a uniform plan under the ægis of some central body such as the British Medical Association. But in any case care must be taken to secure a reasonable reward for the services rendered, and to prevent the intrusion of those who are able to pay ordinary fees. Unless these points be secured there will always remain in such work a tendency to partake of the nature of an immoral contract. No man can retain his self-respect or the respect of others, if he contracts to do work which he knows cannot be properly done at the price, or accepts payment for services which he has no intention of rendering. Yet is not this the very essence of too much contract practice at the present day? "What," says the *Lancet*, "is likely to be the attitude of mind of the medical man when summoned repeatedly to a patient who is paying a few pence when he could afford to pay, and ought to be paying, as many pounds? Does he go prepared to employ all the care and skill that he is capable of, or does he go imbued with the notion of getting through the case as quickly as possible, and thus being 'done' by it as little as may be?" After all, as our contemporary says, "even medical practitioners are human," and we cannot but fear that contract practice, worked as it now is for the benefit of friendly societies and medical aid associations, often brings into play much that is worst in human nature.

